# Supplementary material for: Design, Synthesis, and Biological Evaluation of N-Acylhydrazones and Their Activity Against Leishmania amazonensis Promastigotes
Source: Microorganisms. 2025 Jul 2;13(7):1563. doi: 10.3390/microorganisms13071563 (PMC12298860; doi:10.3390/microorganisms13071563)

PMM 102-17 - Joao Mafra
Op. Eliane

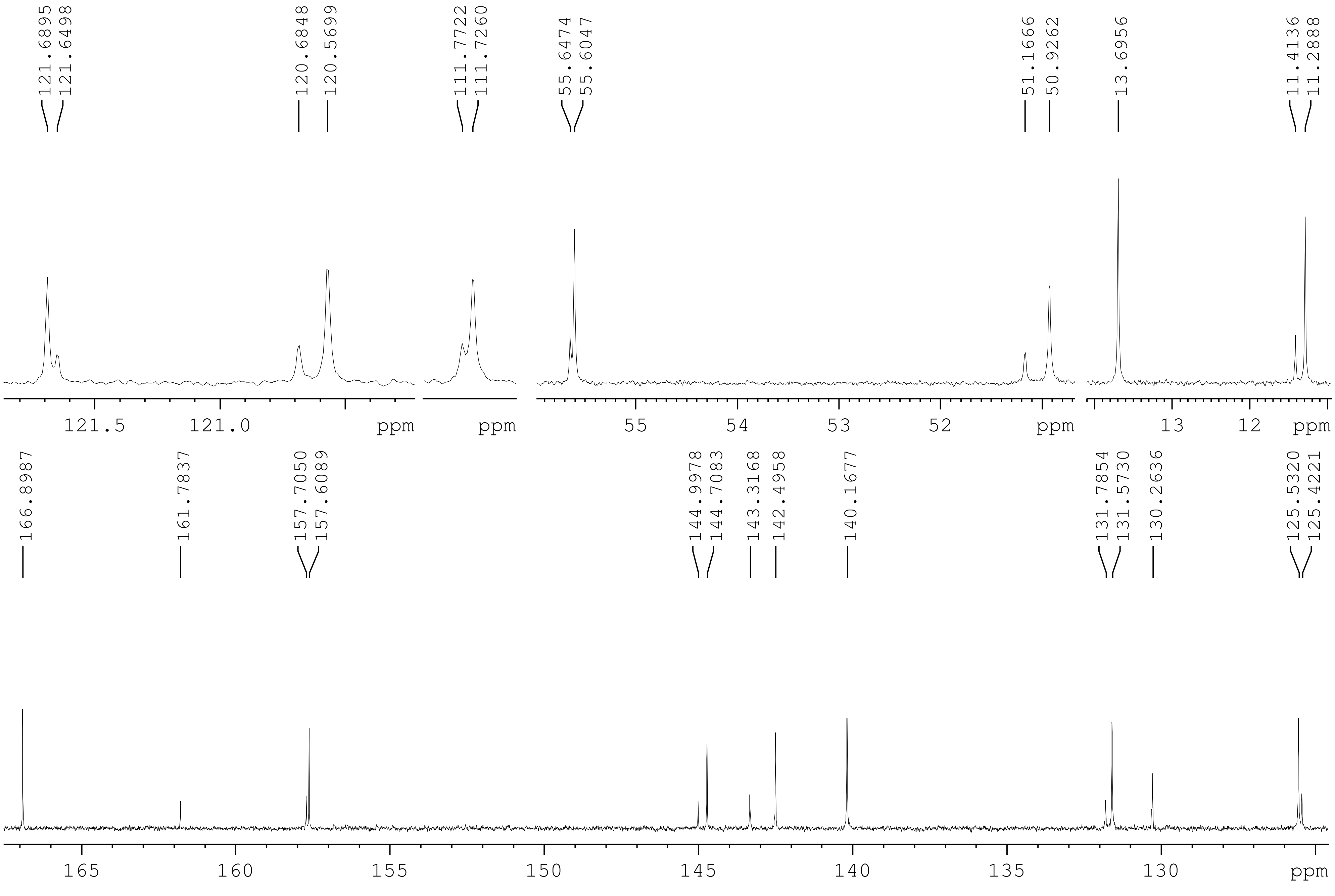

Supplement: Supplementary file 1 [file microorganisms-13-01563-s001.zip › RMN amostras-20241114T155656Z-001.zip!/RMN amostras/composto 12 - PMM 102-17 13Ca.pdf]
